# Metabolomic profiles of myocardial ischemia under treatment with salvianolic acid B

**DOI:** 10.1186/1749-8546-7-6

**Published:** 2012-03-13

**Authors:** Yonghai Lu, Yue Zheng, Xinru Liu, Xu Liang, Saiming Ngai, Tiejun Li, Weidong Zhang

**Affiliations:** 1 Department of Medicinal Chemistry of Nature Product, School of Pharmacy, Second Military Medical University, No. 325 Guohe Road, Shanghai 200433, China; 2 Department of Pharmacology, School of Pharmacy, Second Military Medical University, No. 325 Guohe Road, Shanghai 200433, China; 3 School of Life Sciences, Faculty of Science, The Chinese University of Hong Kong, Hong Kong, China; 4 Institute for Drug and Instrument Control of Health Department, General Logistics Department of the Chinese People's Liberation Army, Beijing 100071, China

## Abstract

**Background:**

*Radix Salvia miltiorrhiza *(*Danshen*) has been used as a principal herb in treating cardiovascular diseases in Chinese medicine. Salvianolic acid B (SA-B), a water-soluble active component of *Danshen*, was found to have anti-myocardial ischemia (anti-MI) effect. This study aims to investigate mechanisms of SA-B on MI.

**Methods:**

Five conventional Western medicines (isosorbide dinitrate, verapamil, propranolol, captopril and trimethazine) with different mechanisms for treating cardiovascular diseases were selected as positive references to compare with SA-B in changing of the metabolomic profiles in MI rats under treatment. Potential mechanisms of SA-B were further investigated in H9C2 cell line.

**Results:**

The metabolomic profiles between SA-B- and propranolol-treated MI rats were similar, since there was a big overlap between the two groups in the PLS-DA score plot. Finally, it was demonstrated that SA-B exhibited a protective effect on MI mainly by decreasing the concentration of cyclic adenosine monophosphate (cAMP) and Ca^2+ ^and inhibiting protein kinase A (PKA).

**Conclusion:**

SA-B and propanolol exhibited similar metabolomic profiles, indicating that the two drugs might have a similar mechanism.

## Background

Myocardial ischemia (MI) is characterized by ischemia in the heart muscle. It is the most common cause of death, and a major reason for hospital admissions [1]. In MI, β-adrenoceptor is generally activated. Noradrenaline binds to β-adrenoceptor to activate GS protein. Then, adenylate cyclase (AC) is activated by GS protein, which makes adenosine triphosphate (ATP) to be cyclic adenosine monophosphate (cAMP). cAMP in turn activates the cAMP-dependent protein kinase A (PKA). This kinase phosphorylates several proteins related to excitation-contraction coupling, such as L-type Ca^2+ ^channel and phospholamban. The phosphorylation of L-type Ca^2+ ^channel causes the Ca^2+ ^influx, leading to stronger muscle contraction. The phosphorylation of phospholamban accelerates Ca^2+ ^uptake into the sarcoplasmic reticulum, increasing the rate and extent of muscle relaxation [2,3].

β-adrenergic blockers such as propranolol [4] could inhibit the activation of β-adrenoceptor and decrease the concentration levels of cAMP, PKA and Ca^2+^, leading to slow heart rate, decreased myocardial contractility, reduced cardiac output, and decreased myocardial oxygen consumption. Besides, Western medicines with other mechanisms of action, such as isosorbide dinitrate (a vasodilator), verapamil (a calcium antagonist), captopril (an angiotensin converting enzyme inhibitor) and trimethazine (a fatty acid oxidation inhibitor), are also commonly used in the prevention and treatment of MI.

In recent years, *radix salvia miltiorrhiza *(*Danshen*) is also widely used in Chinese medicine for the treatment of cardiovascular diseases [5]. *Danshen *preparations in China medicine, including Compound *Danshen *Tablet, Compound *Danshen *Dripping Pill and Compound *Danshen *Granule [6-8] have been developed with interest from pharmaceutical industry.

Lam et al. [9,10] found that the dilator action of *Danshen *on rat femoral artery was primarily produced by the inhibition of Ca^2+ ^influx in the vascular smooth muscle cells. Kim et al. [11] found that *Danshen *could activate the endothelial nitric oxide synthase to induce vasodilation and reduce blood pressure. However, there is no final conclusion about mechanisms of *Danshen*. Thus, most scientists hope to increase understanding of mechanisms of *Danshen *by analyzing active components of *Danshen*. Presently, many active components have been isolated and identified in *Danshen*, such as tanshinone IIA, and salvianolic acid B (SA-B) [12-14].

SA-B, a water-soluble active component of *Danshen*, is effective for the protection of heart from ischemia [15]. Several possible cardio-protective effects were proposed, including augmenting vascular endothelial growth factor (VEGF) expression, promoting angiogenesis, recovering the normal expressions of sarco/endoplasmic reticulum ATPase 2a and phospholamban, and inhibiting the activation of platelet during myocardial ischemia and reperfusion [16-20].

Metabolomics is an emerging technique in the field of "omics" research and it is the systematic study of metabolites and its profile in a biological matrix, such as a cell, organ or organism [21,22]. It aims to pinpoint interesting metabolites that are related to disease or drug treatment. Verhoeckx et al. [23] successfully integrated the transcriptomic, proteomic and metabolomic techniques to characterize inflammatory modulators on the basis of their biological responses. Zilpaterol, a compound originally developed as a β_2_-agonist but later specifically introduced as a growth-promoting agent, showed a pattern of mRNA and lipid expression almost identical to that showed by clenbuterol and salbutamol, β_2_-agonists. In the same way, anti-MI drugs may be categorized into classes based on their mechanisms of action.

In this study, metabolomic approach was used to explore potential mechanisms of SA-B by comparing with five Western medicines (isosorbide dinitrate, verapamil, propranolol, captopril and trimethazine) conventionally used for the treatment of cardiovascular diseases.

## Methods

### Materials

HPLC grade acetonitrile was purchased from JT Baker (NJ, USA). Spectroscopic grade formic acid, leucine enkephalin, dimethyl sulfoxide (DMSO) and 3-(4, 5-dimethylthiazol-2-yl)-2, 5-diphenyltetrazolium bromide were purchased from Sigma/Aldrich (MO, USA). Distilled water was purified "in-house" using a Milli-Q20 system Millipore (MA, USA). SA-B was purchased from the National Institute for the Control of Pharmaceutical and Biological Products (Beijing, China). Isosorbide dinitrate was purchased from Forward Co., Ltd (Shanghai, China). Verapamil was purchased from Shanghai Pharmaceutical (Group) Co., Ltd (Shanghai, China). Propranolol was purchased from Shanghai Xinpashi Pharmaceutical Co., Ltd (Shanghai, China). Captopril was purchased from Shanghai Hengshan Pharmaceutical Factory (Shanghai, China). Trimethazine was purchased from Servier Pharmaceutical Factory (Tianjin, China). Fluo3/AM was purchased from Dojindo Laboratory (Tokyo, Japan). cAMP-Glo™ Assay and PepTag^® ^Non-Radio cAMP-Dependent Protein Kinase Assay were purchased from Promega (Madison, USA).

### Animals and drug administration

Fifty male Sprague-Dawley rats (185-215 g) were purchased from the Slac Laboratory Animal Co., Ltd (Shanghai, China). The animals were housed in stainless steel metabolic cages under controlled conditions of humidity (40-60%), temperature (23-27°C), and a 12 h light-dark cycle. They were allowed free access to food and tap water. MI model was induced by left anterior descending coronary artery ligation [24]. The sham group rats were given no ligation. Electrocardiograms (ECG) were recorded by MPA 2000 Bio-signal Analysis System (Alcott Biotech Co. Ltd., Shanghai, China) to ascertain that the induction of MI was successful. Among 42 MI rats, 36 were arbitrarily selected and divided into six treatment groups (n = 6): SA-B (20 mg/kg/d), isosorbide dinitrate (3 mg/kg/d), verapamil (6 mg/kg/d), propranolol (20 mg/kg/d), captopril (4 mg/kg/d) and trimethazine (6 mg/kg/d). In sham (n = 6) and MI (n = 6) groups, rats were received 0.2 mL of saline each time. Drugs were weighed and crushed into fine powder, which were orally administrated to rats with saline for 7 days after modeling. Rats were fasted overnight but with free access to water before their first administration. Blood samples were collected from ophthalmic venous plexus on the ninth day. The experiment was carried out in accordance with the guidelines of the Committee on the Care and Use of Laboratory Animals of the Institute of Laboratory Animal Resources of Shanghai, China. The Animal Care and Use Committee of Second Military Medical University also approved the study protocol.

### Cell culture

The H9C2 cell line (Catalog No. GNR 5) was obtained from the Cell Bank of the Chinese Academy of Sciences and maintained in Dulbecco's Modified Eagle's Medium (DMEM; GIBCO, Paisley, UK) containing 10% fetal bovine serum (FBS), penicillin-G (100 U/mL) and streptomycin (100 U/mL), and incubated in a humidified CO_2 _incubator (Sanyo, Japan) at 37°C with 5% CO_2_. The cells were seeded at a density of 1 × 10^4^/well in a 96-well plate, and cultured for 12 h for adherence. Cells were pre-treated with propranolol (0.01 mg/mL) or SA-B at concentrations of 0.001, 0.01 and 0.1 mg/mL for 2 h, and the cells were incubated in a Tri-Gas/CO_2 _incubator (Sanyo, Japan) at 37°C with 5% CO_2 _and 95% N_2 _which was anoxic circumstance for 24 h. The cells were incubated in a humidified CO_2 _incubator (Sanyo, Japan) at 37°C with 5% CO_2 _as a control group. Ca^2+^, cAMP and PKA in the cells were measured. SA-B and propranolol were dissolved in DMSO. Appropriate amount of DMSO was also added to control media so that the same final concentration of DMSO (less than 0.1%) can be kept in all groups.

### Metabolomics

A blood sample (1 mL) from each rat was collected and kept in 2.5 mL heparin-coated tubes. These samples were centrifuged at 2789.1 × *g *for 10 min at 4°C, and the supernatants were collected. Acetonitrile (300 μL) was added to the supernatant (100 μL). The mixture was shaken vigorously for 30 s and centrifuged at 9562.5 × *g *for 10 min at 4°C. After centrifugation, the supernatant was analyzed on an ACQUITY™ UPLC system coupled to a Micromass Q-Tof Micro™ (Waters MS Technologies, Manchester, UK) equipped with an electrospray ionization source. A 2.1 mm i.d. × 100 mm ACQUITY™ 1.7 μm column (Waters, Milford, MA, USA) was used. The column was maintained at 45°C. Mobile phase A was formic acid (0.1%) in water; mobile phase B was formic acid (0.1%) in acetonitrile; injection volume was 5 μL; flow rate was 400 μL/min. The gradient duration program was: 0-1.5 min, 5% of B; 1.5-9 min, 5-100% of B; 9-14 min wash with 100% of B; and a 3 min recycle time. The parameters of mass detection were: desolvation gas at 400 L/h; cone gas at 20 L/h; desolvation temperature at 250°C; source temperature at 100°C; capillary voltage at 3000 V; cone voltage at 30 V; collision energy at 5 eV, while it was set at 20 eV in MS/MS mode to identify potential biomarkers. The data acquisition rate was set as 0.4 s, with 0.1 s inter-scan delay. Lock spray was used to calibrate accuracy of mass. Leucine enkephalin was used as the lock mass (m/z 556.2771 in the positive mode, and 554.2615 in the negative mode). Data were collected in the continuum mode and averaged over 10 scans. The lock spray frequency was set at 10 s. Full scan mass range of 50-1000 m/z was acquired.

### Cell viability

Cell viability was assessed by the MTT assay [25]. The cells were treated with SA-B at concentrations of 0.0001, 0.001, 0.01 and 0.1 mg/mL for 24 h. Then, the optical density (OD) values of samples were measured using a microplate reader (Thermo Lab systems, Shanghai, China) at 570 nm (630 nm as a reference).

### Measurement of calcium

The Ca^2+^-sensitive was detected by dyeing with fluo-3/AM [26]. The changes of Ca^2+ ^in cells were examined by flow cytometry (BD Biosciences, USA).

### Measurement of cAMP

The cAMP-Glo™ assay was used to monitor changes in cAMP concentrations [27]. The luminescence was measured by the plate-reading luminometer (Thermo Lab systems, Shanghai, China).

### Determination of PKA activity

The activity of PKA was detected by non-radioactive PepTag PKA assay kit (Promega, Madison, USA) with dye-labeled Kemptide as substrate according to the manufacturer's protocol [28]. The cells were first homogenized in a cold PKA extraction buffer. After centrifugation, the supernatants were collected for the determination.

### Data processing

The spectral data were exported by Micromass MarkerLynx™ applications manager version 4.1 software (Waters Corporation, Milford, MA, USA). The raw data of each sample was normalized to total area to correct for the MS response shift from the first injection to the last injection due to the long duration, overnight or longer, of an LC-MS analysis. The sum of the ion peak area within each sample was set at 10,000. After processing, partial least squares discriminant analysis (PLS-DA) was used for analysis of metabolite profiles, which was performed by the SIMCA-P software version 11 (Umetrics AB, Umeå, Sweden). In cell culture, each assay was performed in triplicate and repeated three times. The data were expressed as mean ± standard deviation (SD). The significance was tested by one-way analyses of variance (ANOVA) of the SPSS 13.0 for Windows (SPSS Inc., Chicago, IL, USA), followed by the Duncan *post hoc *test. *P *values less than 0.05 were considered significant.

## Results and discussion

This study aims to investigate mechanisms of SA-B on MI. Firstly, metabolomics in combination with multivariate statistical analysis was used to display potential mechanisms of SA-B by comparing with five Western medicines (isosorbide dinitrate, verapamil, propranolol, captopril and trimethazine) conventionally used for the treatment of cardiovascular diseases. Secondly, the potential mechanisms of SA-B suggested by the metabolomic study were further investigated in H9C2 cell line.

### Metabolomics

In our previous study [29], a total of 2700 ions were detected in metabolic profile of rats; 160 out of 2700 ions were significantly changed in MI rats as compared with sham rats; 22 out of 160 ions were identified as biomarkers of MI, including 15(S)-HETE, 2',3'-Cyclic AMP, dihydrosphingosine, 5-phosphoribosylamine, phytosphingosine, L-isoleucyl-L-proline, 2',3'-Cyclic GMP, 1-phenylethylamine, thromboxane B2, xanthine, hypoxanthine, inosine, L-homoserine, carnosine, allantoin, L-valine, L-phenylalanine, dihydrobiopterin, 2-oxoisocaproic acid, L-isoleucine, L-tryptophan and glyceraldehydes. In addition, SA-B was also found to be effective for treating MI [8]. The present study revealed the anti-MI effect of SA-B in more detail.

### Anti-myocardial ischemia effect of SA-B

**Figure 1:**
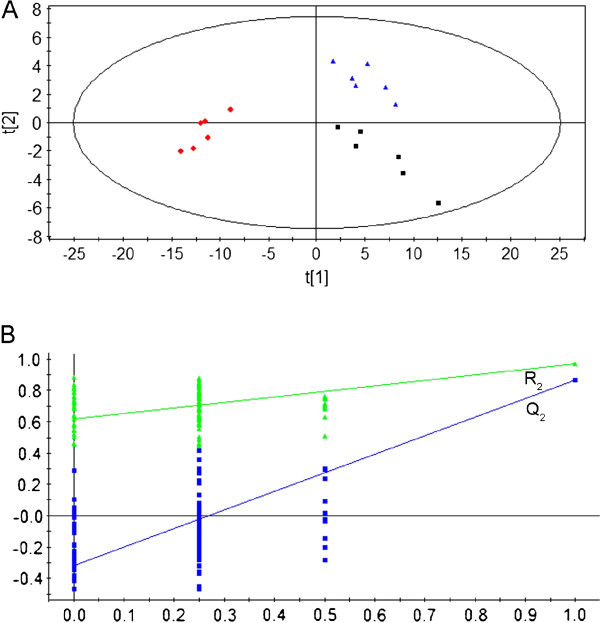
**(a) PLS-DA score plot of the metabolic profiles obtained from 6 sham rats (■), 6 MI rats with no treatment (red diamond symbol), and 6 MI rats with SA-B treatment (blue triangle symbol)**. (R^2^X = 0.798, R^2^Y = 0.975, Q^2 ^= 0.858, A = 4, N = 18, K = 160); (b) Validation plot obtained from permutation test (n = 100). R^2 ^is the explained variance, and Q^2 ^is the predictive ability of the model.

PLS-DA model [29] was built based on the significantly changed ions (Top 160) observed in MI to evaluate the protective effects of SA-B on MI. The PLS-DA score plot (Figure 1A) showed that sham, MI and SA-B-treated rats were classified, and the SA-B-treated rats were much closer to the sham rats than the MI rats, suggesting that SA-B had a positive impact on repairing the abnormal metabolic profiles induced by MI. To validate the result statistically, the PLS-DA model was validated by permutation test (n = 100) (Figure 1B). Generally, Q^2^-intercept in permutation test should be less than 0.05. The intercept value of Q^2 ^for this model was -0.315, which meant no overfitting.

The mean levels of 22 identified MI biomarkers in SA-B-treated rats were calculated to further evaluate the regulations of SA-B. As shown in Table 1, levels of 18 biomarkers in SA-B-treated rats were statistically reversed by compared with those in MI rats (*P *< 0.05, SA-B-treated groups versus MI group) [8]. The result indicated that SA-B could regulate the abnormal metabolites induced by MI.

**Table 1:** Beneficial effects of SA-B and propranolol on MI biomarkers

Biomarkers	Sham group	MI group	SA-B group	Propranolol group	*P *value	
	
	**Mean ± SD**^**a**^	Mean ± SD	Mean ± SD	Mean ± SD	SA-B *vs*. MI	Propranolol *vs*. MI
Allantoin	23.87 ± 4.14	0.00 ± 0.00	20.32 ± 5.36	23.16 ± 8.17	0.000	0.000

1-Phenylethylamine	4.13 ± 1.88	23.66 ± 1.33	12.61 ± 0.96	9.42 ± 1.36	0.001	0.000

Hypoxanthine	0.00 ± 0.00	14.72 ± 14.01	6.68 ± 2.86	0.00 ± 0.00	0.001	0.000

L-Valine	37.98 ± 3.80	13.17 ± 2.14	22.75 ± 4.25	29.41 ± 6.31	0.002	0.001

L-Isoleucine	122.84 ± 21.91	39.18 ± 2.37	92.59 ± 12.26	139.28 ± 26.48	0.001	0.000

Carnosine	16.18 ± 2.70	0.00 ± 0.00	22.13 ± 04.27	19.35 ± 2.34	0.000	0.000

L-isoleucyl-L-Proline	28.69 ± 3.90	79.23 ± 21.02	52.15 ± 14.06	41.32 ± 20.49	0.027	0.011

Phytosphingosine	16.74 ± 1.89	82.93 ± 10.12	32.17 ± 6.29	41.38 ± 5.44	0.000	0.000

Dihydrosphingosine	26.02 ± 0.99	146.98 ± 15.82	52.18 ± 11.40	31.37 ± 7.14	0.001	0.000

2',3'-Cyclic GMP	6.30 ± 0.81	37.05 ± 5.97	15.16 ± 3.49	11.32 ± 2.40	0.001	0.000

2',3'-Cyclic AMP	30.33 ± 1.78	190.63 ± 16.91	153.21 ± 19.48	51.43 ± 8.46	0.008	0.000

Dihydrobiopterin	42.64 ± 4.78	8.23 ± 3.82	19.18 ± 8.31	39.27 ± 8.19	0.015	0.000

Xanthine	0.00 ± 0.00	25.94 ± 6.39	24.13 ± 7.51	0.00 ± 0.00	0.805	0.000

Inosine	0.00 ± 0.00	12.84 ± 3.49	12.03 ± 0.89	0.00 ± 0.00	0.913	0.000

Glyceraldehyde	189.71 ± 13.83	31.23 ± 2.72	99.30 ± 9.47	159.29 ± 12.69	0.000	0.000

L-Phenylalanine	14.65 ± 4.69	0.00 ± 0.00	5.34 ± 2.06	9.28 ± 2.44	0.000	0.000

L-Tryptophan	64.74 ± 12.98	20.06 ± 1.15	77.27 ± 2.40	59.45 ± 5.46	0.000	0.000

2-oxoisocaproic acid	27.85 ± 3.98	6.13 ± 1.23	17.33 ± 3.54	29.75 ± 4.94	0.000	0.000

5-Phosphoribosylamine	87.81 ± 4.96	211.14 ± 15.24	203.47 ± 10.26	129.32 ± 24.94	0.871	0.002

15(S)-HETE	0.00 ± 0.00	243.89 ± 99.98	71.67 ± 4.40	0.00 ± 0.00	0.001	0.000

Thromboxane B2	2.87 ± 1.95	18.27 ± 2.92	11.03 ± 0.64	9.42 ± 3.38	0.002	0.001

^a ^Mean peak areas of MI biomarkers in different groups were calculated to reveal the beneficial effects of SA-B and propranolol; *SD *denotes standard deviation.

### Potential mechanisms of SA-B

**Figure 2:**
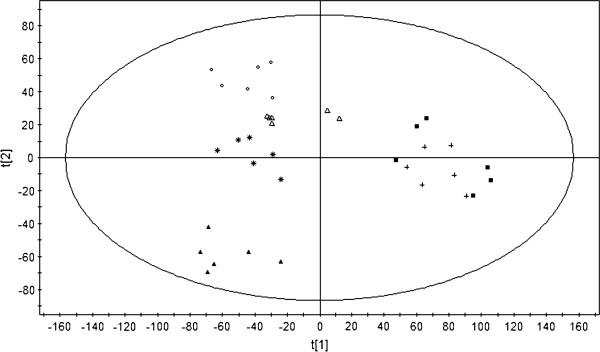
PLS-DA score plot of the metabolic profiles obtained from all drug-treated rats: (+) SA-B, (■) propranolol (Δ) isosorbide dinitrate, (○) trimethazine, (*) verapamil, and (▲) captopril. (R^2^Y = 0.61, R^2^X = 0.833, Q^2 ^= 0.512, A = 5, N = 36, K = 2700).

In order to investigate the mechanisms of SA-B, we selected five Western medicines (isosorbide dinitrate, verapamil, propranolol, captopril and trimethazine) with different mechanisms for treating MI as positive references. PLS-DA model [29] was established based on the global metabolomic profile of MI rats to compare the influences of SA-B and five Western medicines. The PLS-DA score plot (Figure 2) showed five Western medicine-treated groups were separated and the SA-B-treated group overlapped with the propranolol-treated group (each symbol in the plot represents a sample), indicating that the metabolomic profile of SA-B-treated rats was similar with that of propranolol-treated rats. Model validation with permutation test (n = 100) generated intercepts of Q^2 ^= -0.301, which suggested there was no overfitting. The result indicated that SA-B and propanolol might have a similar mechanism in treating MI, since the drugs with the same mechanism showed similar effects on the metabolomic profile [23].

By monitoring the changes of the 22 biomarkers after administration, the regulations of SA-B and propranolol were further compared. As aforementioned, 18 biomarkers were regulated by SA-B (*P *< 0.05, SA-B-treated group versus MI group) (Table 1). Herein, we found that levels of 21 biomarkers were reversed in propranolol-treated rats by compared with those in MI rats (*P *< 0.05, propranolol-treated group versus MI group) (Table 1) [29]. The results further suggested that SA-B and propranolol had similar regulations on biomarkers of MI rats, indicating they might have a similar mechanism.

### Validation of mechanisms of SA-B in H2C2 cell line

As aforementioned, propranolol [4] could inhibit the activation of β-adrenoceptor and decrease the concentration levels of cAMP, PKA and Ca^2+^, leading to slow heart rate, decreased myocardial contractility, reduced cardiac output, and decreased myocardial oxygen consumption. In order to prove the propranolol-like effect of SA-B, we tested whether SA-B could block β-adrenoceptor directly. Unfortunately, specific binding of SA-B to β-adrenoceptor was not found in our early study (not published). In this study, we focused on the influences of SA-B and propranolol on the downstream regulation in β-adrenoceptor signaling pathway. The effects of SA-B on the levels of on cAMP, PKA and Ca^2+ ^in cells were investigated by H9C2 cell line, which is a commercially available myogenic cell line derived from embryonic rat heart ventricle [30] and is widely accepted as a substitute for rat cardiomyocyte in in vitro study [31].

**Figure 3:**
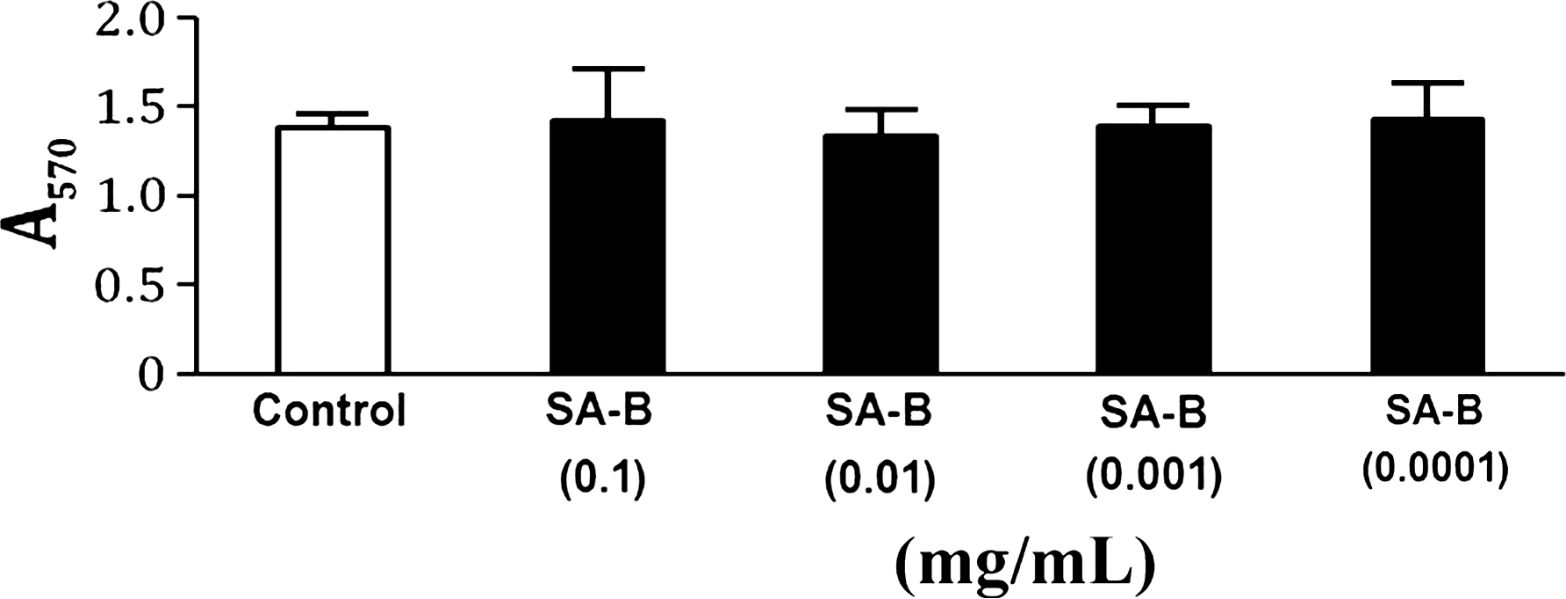
**Effects of SA-B on H9C2 cell viability**.

**Figure 4:**
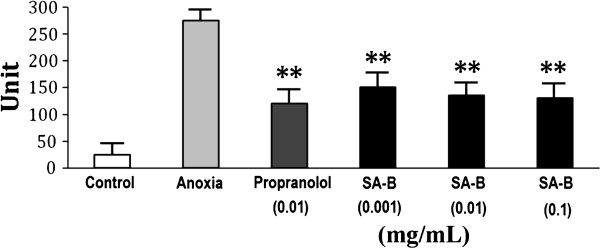
**Effects of SA-B on the cellular calcium concentration in H9C2 cells**. ** *P *< 0.01 compared with anoxia.

**Figure 5:**
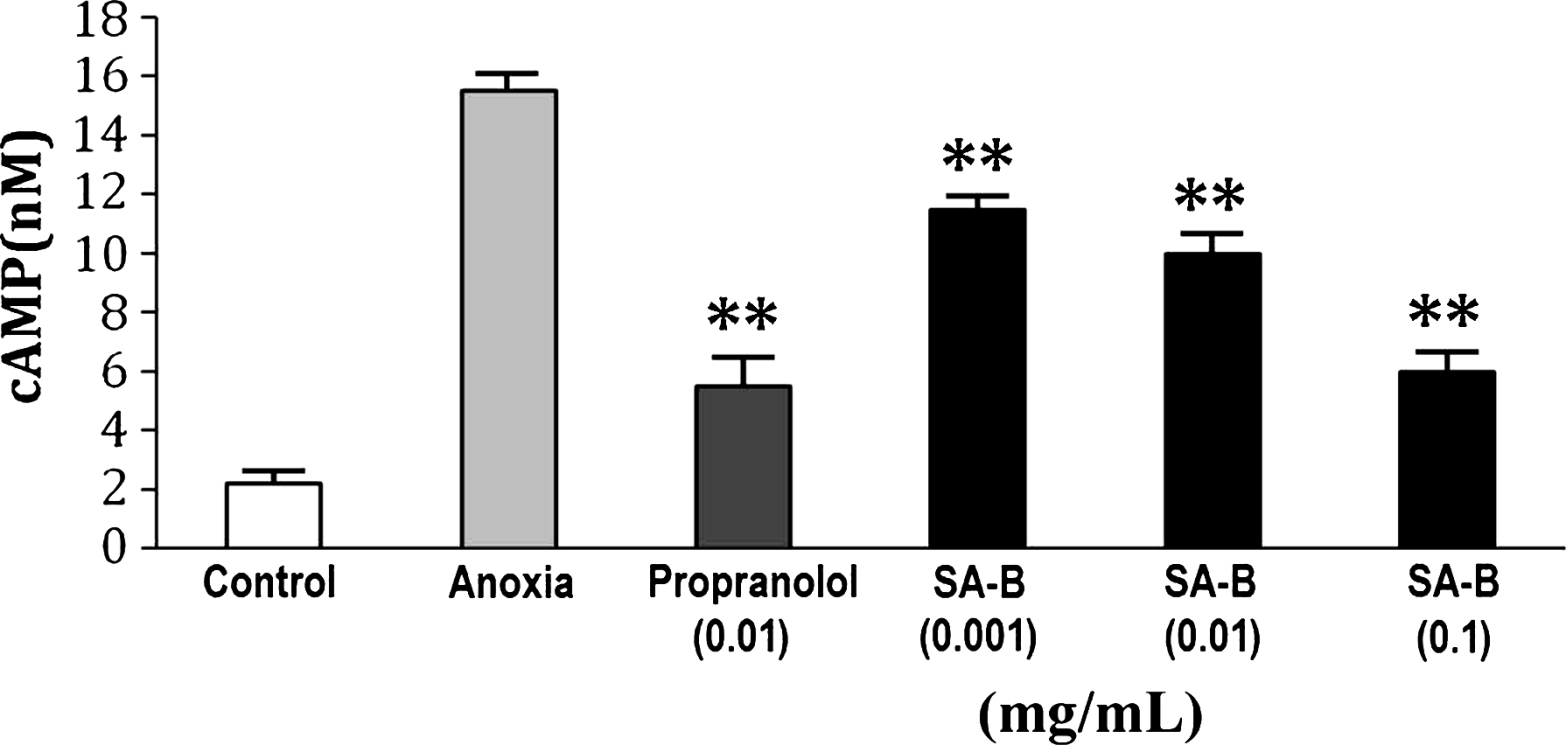
**Effects of SA-B on the levels of cAMP in H9C2 cells**. ** *P *< 0.01 compared with anoxia.

**Figure 6:**
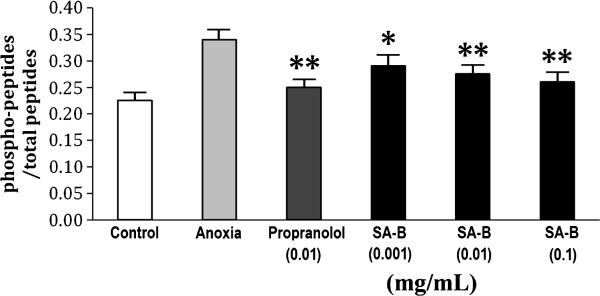
**Effects of SA-B on the PKA activity in H9C2 cell**. ** *P *< 0.01 compared with anoxia.

The viability of H9C2 cells was not affected significantly by pretreatment with SA-B up to 0.1 mg/mL for 24 h (Figure 3), indicating that SA-B did not cause damage to H9C2 cells. The results of Ca^2+^, cAMP and PKA assays showed that SA-B significantly decreased Ca^2+ ^concentration in anoxic H9C2 cells (Figure 4). We suggested that SA-B might have a strong effect on the regulation of Ca^2+ ^concentration even at a very low dosage (0.001 mg/mL). We also found that both SA-B and proprannolol significantly inhibited the increased levels of cAMP induced by anoxia (Figure 5). SA-B decreased cAMP concentration in a concentration-dependent manner. As shown in Figure 6, PKA activity was increased in anoxic cells, but significantly decreased by SA-B and proprannolol treatment. In addition, there was also no significant difference among these three SA-B-treated groups. These results showed that SA-B could decrease the concentration of Ca^2+ ^and inhibit the activation of PKA induced by anoxia at a very low dosage (0.001 mg/mL), but required a high dosage (0.1 mg/mL) to decrease the concentration of cAMP. The cell experiments demonstrated that SA-B had a similar role in regulation of concentrations of Ca^2+^, cAMP and activity of PKA as proprannolol.

Our previous studies suggested that the metabolomic profile was changed in MI rats [29], and SA-B could reverse the levels of most MI biomarkers [8]. The present study indicated that SA-B- and propanolol-treated rats had similar metabolomic profiles, which suggested that the two drugs might have a similar mechanism in treating MI, since the drugs with the same mechanism showed similar effects on the metabolomic profile. The hypothesis that SA-B and propranolol had a similar mechanism in treating MI was supported by these experiments. Although no evidence showed that SA-B could block β-adrenoceptor directly, the cell experiments demonstrated that SA-B showed a similar role in regulation of concentrations of Ca^2+^, cAMP and activity of PKA as proprannolol. While the drug action of the metabolites of SA-B is still under investigation, our results suggest that metabolomics is a good analytical method for understanding mechanisms of SA-B.

## Conclusions

SA-B is a water-soluble active component of *Danshen*, and is potentially effective for the protection of heart from ischemia-reperfusion. Metabolomic results showed that SA-B and propranolol had similar effects on metabolic profiles of MI rats. The cell experiments demonstrated that SA-B had protective effects on MI mainly by decreasing Ca^2+ ^and cAMP and inhibiting the activation of PKA. This study indicates that SA-B and propanolol might have a similar mechanism in treating MI.

## Abbreviations

MI: myocardial ischemia; AC: Adenylate cyclase; ATP: adenosine triphosphate; cAMP: cyclic adenosine monophosphate; PKA: protein kinase A; SA-B: salvianolic acid B; VEGF: vascular endothelial growth factor; DMSO: dimethyl sulfoxide; ECG: electrocardiograms; DMEM: Dulbecco's Modified Eagle's Medium; FBS: fetal bovine serum; OD: optical density value; PLS-DA: partial least squares discriminant analysis; SD: standard deviation; ANOVA: one-way analyses of variance.

## Competing interests

The authors declare that they have no competing interests.

## Authors' contributions

YHL, YZ, TJL and WDZ designed the study. YHL conducted the metabolomic study and wrote the manuscript. YZ assisted in the cell experiments and in writing the manuscript. XRL and XL assisted in writing the manuscript. SMN, TJL and WDZ amended the manuscript. All authors read and approved the final manuscript.
